# Profession-specific working conditions, burnout, engagement, and turnover intention: the case of Hungarian social workers

**DOI:** 10.3389/fsoc.2024.1487367

**Published:** 2024-12-04

**Authors:** Ágnes Győri, Szilvia Ádám

**Affiliations:** ^1^HUN-REN Centre for Social Sciences, Institute for Sociology, Budapest, Hungary; ^2^Semmelweis University, Health Services Management Training Centre, Budapest, Hungary

**Keywords:** burnout, engagement, profession-specific working conditions, turnover intention, social workers

## Abstract

**Introduction:**

This study investigated the interactions of profession-specific working conditions, burnout, engagement, and turnover intent among social workers in Hungary. Research on turnover among employees in human services occupations often overlooks the mechanism linking professional-specific factors with turnover intention.

**Methods:**

Using a multistage stratified sampling method and cross-sectional design with a random sample of 664 participants, data were collected through computer-assisted personal interviews.

**Results:**

The findings revealed that poor working environments, workplace struggles, and profession-specific factors, such as challenges stemming from clients' difficult life situations, difficulties with client engagement, cultural disparities, and fieldwork-related challenges, significantly influenced turnover intentions. These factors not only directly affected professionals' intentions to leave but also had indirect effects through burnout and work engagement.

**Discussion:**

Our findings highlighted the importance of addressing both general and profession-specific working conditions to reduce turnover intention among social workers. Key challenges included clients' difficult life situations, cooperation with clients, cultural differences, and fieldwork difficulties. Overall, preparing social workers for real-life situations and related conflicts should be incorporated into their training.

## 1 Introduction

Turnover and skills shortages in the social sector are pervasive issues across Europe. According to the latest Social Work Employer Survey, 34% of social organizations reported significant staff shortages, particularly in over 10% of vacant roles (EPSU, 2022). In Hungary, ~57,000 professionals are employed in institutions providing basic and specialized social services, with nearly 3,600 positions remaining vacant (Hungarian Central Statistical Office, 2023). A particularly alarming shortage exists in child protection services, where 25–35% of certain positions, such as psychologists and child-protection clerks, remain vacant (Balogh et al., 2019). Additionally, a 2021 survey found that 32% of social sector workers were planned to leave their jobs during the winter of 2021 (Gyarmati, 2021).

High turnover in social care organizations leads to numerous operational challenges, including rising costs and a decline in care quality, which ultimately affects service delivery (Hom et al., 2017; Pharris et al., 2022). Moreover, turnover negatively impacts both clients and professionals, contributing to lower service quality, reduced client trust, and increased anxiety among professionals (De Croon et al., 2004). This underscores the need to understand the correlates of turnover intentions in social work.

A growing body of literature points to high levels of stress and burnout among social workers as key contributors to turnover intention and actual turnover (Jia and Li, 2021; Kim and Stoner, 2008; Mercado et al., 2022; Ravalier et al., 2021). Hungarian social professionals, in particular, face challenging working conditions, including low salaries, high workloads, and limited resources, all of which are associated with an increased risk of burnout and turnover intentions (Kopasz et al., 2024). In fact, a significant portion of social workers in Hungary is dealing with excessive caseloads, with some managing double the expected number of families due to capacity issues (Husz et al., 2020). Despite slight improvements in material conditions, including a 20% pay raise in 2022, their salaries remain notably low, contributing to demotivation and increased turnover (Czibere and Mester, 2020).

### 1.1 Literature review

In general, employee turnover intent is defined as the probability of leaving the organization soon as assessed by the employees themselves (Tham, 2007). The literature considers turnover intentions to be an immediate precursor of turnover, and several studies showed that employee turnover intentions predicted personnel turnover rates (Lee and Whitford, 2007). Therefore, turnover intention has been utilized in empirical research as a proxy for personnel turnover (Lambert et al., 2001; Yan et al., 2021).

Work-related factors, such as workplace climate, organizational culture, financial compensation, and job satisfaction have been recognized as critical component of turnover intention (Li et al., 2016; Wang et al., 2021). A meta-analysis of empirical studies among social workers and other human services employees by Mor Barak et al. (2001) found that factors influencing professionals' intention to leave the workplace can be classified into three categories: (1) demographic factors, (2) professional perceptions, and (3) organizational settings. Demographic factors pertain to aspects of personal attributes (e.g., age, sex, and tenure with the organization) and work-related background (e.g., social work education). Professional perceptions include organizational and professional commitment, burnout, and job satisfaction. Organizational settings encompass fair compensation (income or benefits) and organizational culture, including support from coworkers and supervisors, cooperative team-based interaction, and physical comfort (Mor Barak et al., 2001).

Research on the turnover of social workers suggests that rather than demographic factors, the main predictors of professionals' intention to quit are circumstances related to work and organization (Kim and Stoner, 2008; Smith and Shields, 2013). Some research also highlights the importance of good communication (communication culture) in retaining employees in the social sector, including fair and humane conduct, interpersonal justice, polite and fair treatment, and respectful behavior in the workplace (Wang et al., 2021).

Burnout has been identified as a significant correlate of turnover intention and turnover itself among social workers (Alarcon, 2011; Kaiser et al., 2020; Kim and Stoner, 2008). Burnout is a syndrome that develops in the occupational setting (Maslach et al., 2001). According to Cherniss (1980), it is a process where specialists' attitudes and behaviors change negatively under the influence of work-related stress. Burnout is a serious problem across different professions, especially in the human services sector. Several studies highlight higher stress and burnout levels among social workers compared to other human service occupations due to their exposure to a various workplace events that can negatively impact mental health (Gyori et al., 2023; Kim and Stoner, 2008).

Factors contributing to burnout include high workloads and job demands, lack of job control, threats of psychological and physical abuse, and working with vulnerable people who have experienced trauma or stressful life events (Ertas, 2015). These are familiar challenges in the social work field. Some research shows child protection professionals face a higher risk of burnout than those in other social services fields (Conrad and Kellar-Guenther, 2006; Gyori and Perpék, 2021). Researchers link this tochild protection's peculiarities and a higher likelihood of developing compassion fatigue, also known as secondary traumatic stress.

Additionally, frontline social workers tend to experience more burnout than other groups, such as those in higher-level positions (Cho and Song, 2017). Some studies also connect burnout among social workers to pressures related to their professional roles and organizational role conflict (Ravalier et al., 2021). Social work professionals encounter a variety of legitimate yet conflicting expectations, such as clients seeking empathy and understanding of their problems, while supervisors demand strict compliance with rules and administrative precision. These conflicting demands can result in tensions with clients. Research on social professionals consistently shows that navigating these roles contributes significantly to emotional exhaustion and the development of impersonal attitudes (Hongfei et al., 2023; Zheng et al., 2022).

Despite experiencing high levels of psychological and emotional strain, social workers appear to find their work rewarding and important (Mandell et al., 2013). Their idealism and commitment to working with children and families serve as sources of motivation, compassion satisfaction, and engagement, as they see the impact of their help (Acker, 2010). In contrast to burnout, work engagement is defined as a positive affective-motivational state associated with wellbeing at work characterized by high levels of energy and measured by vigor, dedication, and absorption (Schaufeli and Bakker, 2004). Engaged employees demonstrate enthusiasm and emotional connections with their work, feeling suited of job demands, leading to lower turnover intentions (Nilsen et al., 2023; Park and Pierce, 2020). Work engagement reduces the likelihood of turnover and is generally associated with improved mental and physical health, as well as work performance (Bakker and Demerouti, 2016).

Although the relationship between work characteristics, burnout, engagement, and turnover intention has been widely studied among healthcare workers, our knowledge on this topic is limited as it relates to social workers. Studies involving nurses and doctors have shown that low job satisfaction significantly contributes to healthcare staff burnout, and those experiencing high levels of burnout are more likely to intend to leave their job (Scanlan and Still, 2013; Wang et al., 2020). It has also been confirmed that burnout and work engagement mediate the relationship between job resources and mobility intention, buffering the negative impact (Margaret and Ranjit, 2013; Ofei-Dodoo et al., 2020; Shemueli et al., 2016).

However, research on the turnover of social work professionals often overlooks the mechanisms linking occupational-specific working conditions with turnover intention. The present study aims to fill this gap by (1) exploring the relationships between profession-specific and general work-related factors and turnover intention among Hungarian social workers, and (2) testing the extent to which job burnout and work engagement mediate the relationship between working conditions and turnover intention. By addressing these research questions, the study seeks to contribute to a deeper understanding of how working conditions affect turnover in the social sector and propose strategies to alleviate the shortage of social work professionals. The novelty of our research is also to test established relationships in a new, understudied context. In particular, it will provide insight into the dynamics of turnover intention among social workers in a country with distinctive socio-economic challenges and limited research on this topic. Furthermore, the study will have practical implications for managerial practices in Hungary, where high turnover in social sector is a pressing issue. The findings could inform strategies for improving working conditions, reducing burnout, and enhancing work engagement to reduce turnover intentions in the Hungarian context.

## 2 Method

### 2.1 Data

The data source used for the present analysis is a cross-sectional survey of social workers in Hungary, conducted between May and November 2022. Based on the number of social workers in Hungary, the tolerated margin of error (5%), the confidence level (95%), and expected response rate of at least 80%, a sample of 664 participants was randomly selected from the registries held by the Hungarian Central Statistical Office, reflecting the status of social workers as of 31st December, 2021. The sample was representative for (1) type of specialty of social care (basic social services, family and child welfare services, specialized care, child protection), (2) type of provider (state, municipality, religious organizations, and other non-profit or for-profit organizations), (3) location (territorial units for statistics, NUTS-2 regions), (4) age, and (5) gender. A computer-assisted personal interview (CAPI) was conducted with the sampled social workers. Participants who declined participation were automatically replaced to ensure final sample size. Following data cleaning, we reviewed the distribution of the sample and applied correction weighting to counteract slight deviations from the expected distribution and maintain representativeness. The average sample weight was 1, and the total range was 0.653–1.100.

The study was approved by the Center for Social Sciences Research Ethics Board (code of ethics: TK-20/2021). Participation in the study was voluntary informed consent was obtained before the commencement of the survey.

### 2.2 Measurement

#### 2.2.1 Turnover intention

The turnover intention was measured by the Roodt's Turnover Intention Scale (TIS) using its abridged 6-item version (TIS-6), which is considered by the literature to be a reliable measurement tool for predicting actual fluctuation (Bothma and Roodt, 2013; Muliawan et al., 2009). Using the items of a five-point Likert scale (ranging from “strongly disagree” to “strongly agree”), respondents can indicate how often and with what certainty they perceive the statements listed. The statements relate to 9 months prior to the survey. Some items on the scale include, for example “How often have you considered leaving your job?” or “How likely are you to accept another job at the same compensation level if offered?” The total score of the six items was calculated and defined as the turnover intention score, ranging from 6 to 30, higher scores indicating stronger turnover intention. In this study, the scale demonstrated good internal consistency, with a Cronbach's alpha of 0.804.

Before this study, no Hungarian version of the TIS existed, so translation and counter-translation work were necessary for cultural adaptation purposes. The Hungarian version of TIS was prepared based on international recommendations. The original TIS scale in English was translated into Hungarian by three researchers of the research project for producing the first version, who developed a common version based on the three translations. This version was a counter-translation made by a native-speaker bilingual translator, and there was no substantive difference in meaning between the two versions. The final version was piloted involving 30 people. Based on their opinion, we have made a few more minor changes. Subsequently, we included the questionnaire in our sample, along with other questionnaires.

#### 2.2.2 General and occupational-specific working conditions

The independent variable was characteristics of the social profession with regard to working conditions. The general and profession-specific working conditions were assessed using a multifacted instrument created based on previous literature (Hackman and Oldham, 1975; Lennon, 1994; Munch-Hansen et al., 2008). From one point of view, these statements were aimed at exploring factors of general workplace and work environment yet, from another point of view, indicators that are occupational-specific of social profession, for example problems of cooperation and interaction with clients, management of cultural differences, and factors related to difficulties in fieldwork. The final measures comprised four subscales including attitudes toward the poor working environment with eight items (physical and infrastructural working conditions, support from supervisors, support from colleagues, autonomy, professional development, material rewards, and moral esteem), workplace struggles with six items (workplace task in which they are not proficient, non-essential tasks, often changing rules, unclearness rules or regulations, work-family conflict, and role conflicts), clients-related challenges with five items (clients' ability to cooperate/behavior, clients' special culture, clients' social situation, only short-term assistance to the needy), furthermore fieldwork-related difficulties with three items (too much travel, unsafe field, and travel to difficult to reach places). To ensure a balanced approach, we included statements with both positive and negative evaluations. During the analysis, we reversed the direction of statements with opposite value content so that higher scores consistently reflect a negative attitude toward the statements. Respondents were able to rate the extent to which statements are characteristic of them on a four-point Likert scale (1 – not typical at all; 4 – fully typical). For the four subscales, mean scores were calculated. Cronbach's alphas were 0.889, 0.862, 0.871, and 0.795, respectively, indicating high levels of reliability.

#### 2.2.3 Burnout

Burnout was measured using the Hungarian version of the Mini version of Oldenburg Burnout Inventory (Demerouti et al., 2003), which was validated by Ádám et al. (2020) and used in a previous Hungarian study. The questionnaire measures burnout on two subscales: five items measured exhaustion, which is work-related physical and emotional fatigue (for example, “There are days when I feel tired before I arrive at work”), and five measured disengagement, which contains a lack of motivation in work and a high degree of depersonalization (for example, “Lately, I tend to think less at work and do my job almost mechanically”). All items are scored on a four-point Likert scale, ranging from 1 (“totally disagree”) to 4 (“totally agree”). Both subscales showed acceptable internal consistency: Cronbach's alphas were 0.822 and 0.717, respectively.

#### 2.2.4 Work engagement

The 17-item Utrecht Work Engagement Scale (Schaufeli et al., 2002) was used to measure social workers' engagement, which was used in previous studies among Hungarian professionals in human services sector (e.g., Ádám and Hazag, 2013). The Scale measures three dimensions of work engagement: six items measure vigor (for example, “At my work, I feel bursting with energy”), five measure dedication (for example, “I find the work that I do full of meaning and purpose”), and six measure absorption (for example, “It is difficult to detach myself from my job”). The items were evaluated on seven-point scales scored from 0 (“never”) to 6 (“always”). The three subscales' Cronbach's alphas were 0.886, 0.918, and 0.883, respectively in this study.

#### 2.2.5 Covariables

We also used in the analysis several covariates regarding the social workers' sociodemographic and professional information. Sociodemographic variables included gender, age, marital status (dummy variable: married, single/divorced/widowed), subjective income (categorical variable: can cover usual expenses with difficulties, they can cover them relatively easily and easily or very easily), and education background (social work or related degree, e.g., social policy or sociology). Professional information included experience of social work (year), job position (dummy variable: frontline social worker, middle manager or head of institution), type of specialist area of care (categorical variable: family and child welfare, child protection, social basic services, and specialized social care), atypical work schedule (yes/not), and overtime (above 10 h per week).

### 2.3 Statistical analysis

First, we calculated frequency (N) and percentage (%) statistics to describe the socio-demographic and professional characteristics of the participants. Second, we studied descriptive statistics (Mean ± SD) and categorical variables (N, %) and computed Pearson correlation coefficients. Next, we employed structural equation modeling (SEM) to verify the path and synthetic relationships among general and job-specific working conditions, burnout, engagement, and turnover intention. SEM is an optimal technique for test the indirect effects on pre-assumed causal relationships, revealing how variables may influence turnover through other factors. Mediation analysis, which is part of structural equation analysis, was used to analyze the relationships between exposure, outcome, and mediating variables. Maximum likelihood estimation (MLE) was used to estimate the parameters in the SEM. Finally, we performed hierarchical multiple linear regression to analyze the influencing predictors of social workers' turnover intention, and to assess the unique contribution of different groups of predictors to exit turnover. This dual approach ensures that our findings are statistically rigorous and practically relevant, offering clear insights into the most critical predictors of turnover. Multicollinearity among the explanatory variables was assessed using the variance inflation factor (VIF). All statistical analyses were performed using Stata 16.0 software, and a *P*-value < 0.05 was considered statistically significant.

## 3 Results

### 3.1 Characteristics of study participants

The majority of participants were female (85.7%) and in married or common-law relationships (73.6%). The average age was 43.5 years (SD: 10.3). Socioeconomic status was assessed based on subjective income: 35.9% of participants reported experiencing difficulties covering usual expenses, 36.2% reported minor difficulties, and 27.9% reported covering expenses relatively easily or very easily. For the educational level, the vast majority (77.6%) had a higher social education: 48% of them graduated from college while 30% had a university degree. They had been working in the social work profession for an average of 12.7 years (SD: 8.7). Most of the participants (70.6%) work as a firstline social worker, one-sixth (17.6%) as middle managers and another 10th (11.7%) as heads of institutions. Almost the same proportion of participants had been employed in institutions providing basic social services (34.9%) and family and child welfare (32.7%), while a quarter (24.1%) of them worked in specialized social care institutions and 8.3% in child protection care (including those working in foster care networks and children's homes). 10.4% of respondents regularly work atypical hours (night/weekend shifts), and over a third (36.3%) work more than 10 h of overtime per week.

### 3.2 Descriptive and bivariate correlations

Table 1 shows the descriptive statistics of the study variables, including mean scores, standard deviations, and correlations. The average score on the 6-item turnover intention scale is 15.2 (SD = 5.1, min 6, max 27). If the total score given for TIS items is divided into two categories applying the cut-off of 18, following the recommendation of Bothma and Roodt (2013), it is evident that 30.0% of participants are characterized by a serious mobility intention, i.e., a conscious and purposeful intention to leave the workplace.

**Table 1:** Descriptive statistisc and correlations.

	**Subscales**	**M**	**SD**	**CR**	**AVE**	**Correlation**	***P*-value**
Working conditions	Poor working environment	3.01	0.68	0.916	0.812	0.329	<0.001
	Workplace struggles	3.18	0.97	0.865	0.672	0.313	<0.001
	Clients-related challenges	3.24	0.98	0.908	0.712	0.294	<0.001
	Fieldwork-related difficulties	2.77	0.91	0.780	0.548	0.226	<0.001
Burnout	Exhaustion	12.81	3.15	0.934	0.826	0.454	<0.001
	Disengagement	10.91	2.70	0.889	0.782	0.451	<0.001
Work engagement	Vigor	4.98	1.32	0.729	0.517	−0.522	<0.001
	Dedication	5.03	1.51	0.945	0.828	−0.501	<0.001
	Absorption	4.82	1.42	0.865	0.642	−0.438	<0.001
Turnover intention	-	15.26	5.15	0.904	0.693	-	-

M, Mean; SD, Standard Deviation; CR, Composite Reliability; AVE, Average Variance Extracted; Correlation, Pearson correlation coefficient with turnover intention.

In terms of working conditions—combining the typical and fully characteristic categories—it emerges that among the items of the working environment subscale, the highest proportion of respondents (62.1%) consider salary, benefits for work inadequate, but there is also a high proportion of those who report a lack of moral recognition (47.3%), while the smallest proportion (11.2%) consider the support of employees inadequate. Among the items of the workplace struggles subscale, the proportion of those reporting too much administration is outstandingly high (49.1%) and the proportion of those reporting difficulties due to changes in rules/regulations affecting work is also high (43.2%), while the proportion of those reporting conflicts of roles due to the joint performance of assisting and authority roles is the lowest (16.8%). Among the items of the clients-related challenges subscale, the proportion of clients' specific culture reporting difficulties due to their specific culture is the highest (40.8%), but the proportion of those who do not consider the cooperation of beneficiaries to be adequate is also high (33.9%). The items on the fieldwork-related difficulties subscale are considered very similar by respondents: between 15.8 and 16.5% of respondents find it difficult to travel a lot, unsafe terrain and travel to hard-to-reach places.

The average score for the exhaustion dimension of burnout in our sample is 12.8 (SD = 3.1) and the average score for disengagement is 10.9 (SD = 2.7). Based on international standards (Peterson et al., 2008), a significant proportion of the social professionals examined, 67.5%, reach the burned-out range on the exhaustion dimension (above ≥2.25), while it is slightly lower on the disengagement dimension (55.1% above ≥2.51).

Looking at respondents' commitment to work, based on the score limits proposed by Schaufeli and Bakker (2003), 11.7% reported low commitment to work overall: one-eighth (12.5%) had low vigor, a seventh (14.6%) had low dedication and one-tenth (9.9%) had low absorption.

Significant correlations were found between all the variables studied. Turnover intention was significantly positively correlated with each subscale of working conditions and burnout, and negatively associated with each subscale of work engagement. Out of the four subscales of working conditions, turnover intention had the strongest association with working environment (*r* = 0.329) and the weakest association with fieldwork-related difficulties (*r* = 0.226). Both subscales of burnout syndrome were similarly strongly associated with turnover intention (*r* = 0.454 and *r* = 0.451). The correlations between turnover intention and the three subscales of work engagement were as follows: −0.522 for vigor, −0.501 for dedication, and −0.438 for absorption. Additionally, the composite reliability was found to be good (CR > 0.700), and the average variance extracted (AVE > 0.500) indicated acceptable discriminant validity for all latent variables.

### 3.3 Path relationship between working conditions, burnout, engagement, and turnover intention

**Figure 1:**
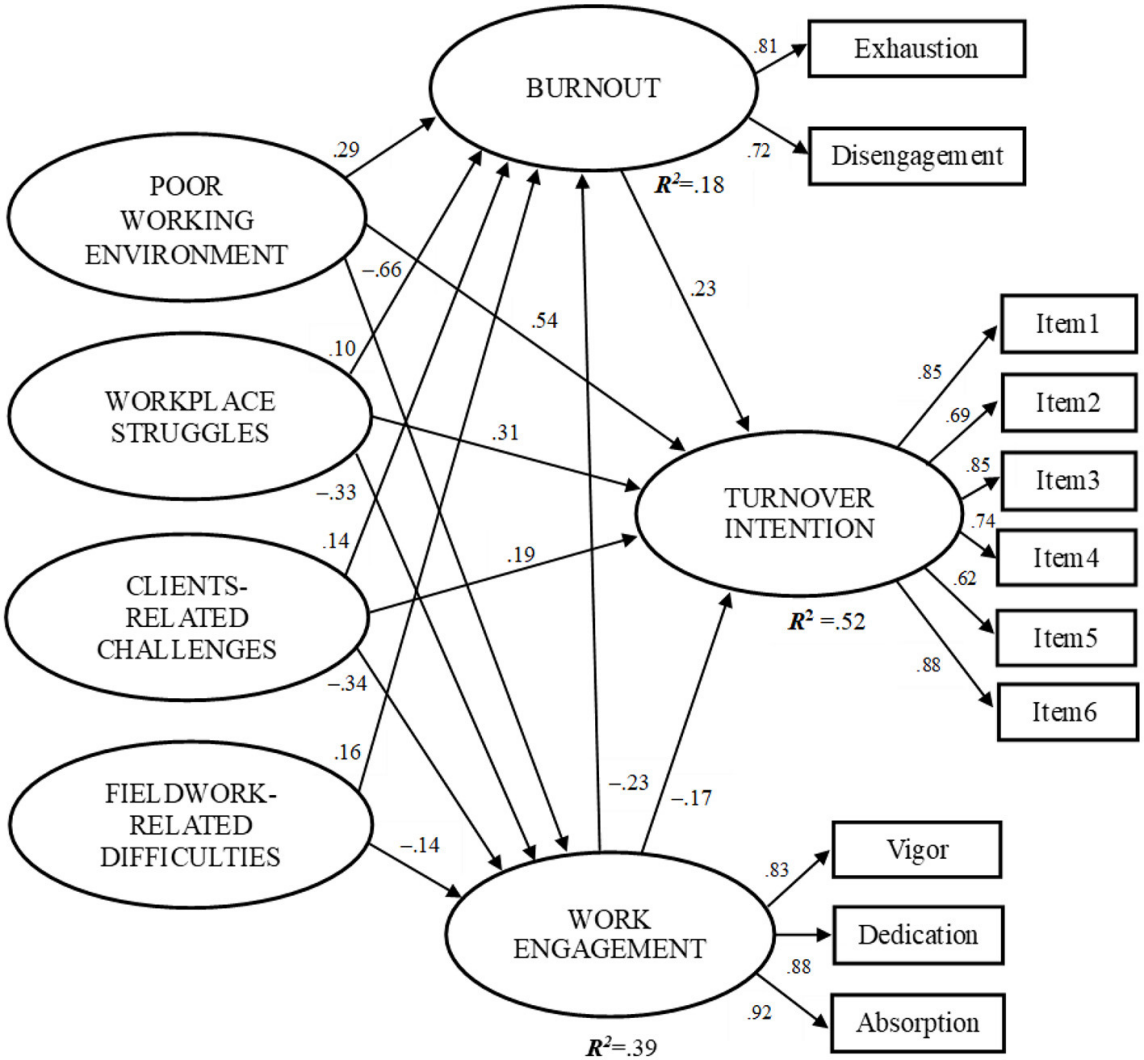
Results of the structural equation model. All coefficients are significant (*p* < 0.05). Standardized regression coefficients are reported.

Models were built to estimate the relationships between general and profession-specific working conditions, burnout, work engagement, and turnover intention. Paths with non-significant coefficients were removed one at a time until only significant paths remained in the model. The final model is presented in Figure 1. All path coefficients are statistically significant at *p* < 0.05. Standardized direct and total indirect effects, adjusted for gender, age, marital status, subjective income situation, social work degree, social work experience, job position, type of specialist care area, atypical work schedule, and overtime, are detailed in Table 2. The model fit indices were as follows: χ^2^/df = 2.205, CFI = 0.937, TLI = 0.915, SRMR = 0.042, and RMSEA = 0.044 with a 95% confidence interval (CI) of 0.038–0.049, indicating a good model fit. The variables accounted for 52.3% of the variance in turnover intention among the social workers in this study.

**Table 2:** Summary of the structural equation model (standardized direct and indirect effects).

	**Standardized coefficient**	**Standard error**	***P* > |z|**	**95% CI**	
				**Lower**	**Upper**
**Direct effects**					
Turnover intention <– Poor working environment	0.540	0.067	0.000	0.407	0.672
Turnover intention <– Workplace struggles	0.313	0.054	0.000	0.206	0.420
Turnover intention <– Clients-related challenges	0.191	0.054	0.048	0.078	0.217
Turnover intention <– Burnout	0.238	0.062	0.000	0.116	0.360
Turnover intention <– Work engagement	−0.174	0.028	0.000	−0.231	−0.118
Burnout <– Poor working environment	0.296	0.054	0.000	0.189	0.401
Burnout <– Workplace struggles	0.104	0.046	0.025	0.012	0.196
Burnout <– Clients-related challenges	0.145	0.049	0.003	0.049	0.241
Burnout <– Fieldwork-related difficulties	0.162	0.040	0.002	0.012	0.285
Burnout <– Work engagement	−0.233	0.034	0.000	−0.425	−0.198
Work engagement <– Poor working environment	−0.660	0.097	0.000	−0.852	−0.468
Work engagement <– Workplace struggles	−0.336	0.085	0.000	−0.503	−0.168
Work engagement <– Clients-related challenges	−0.348	0.095	0.000	−0.526	−0.150
Work engagement <– Fieldwork-related difficulties	−0.145	0.028	0.004	−0.239	−0.099
**Indirect effects**					
Poor working environment —> Burnout —> Turnover intention	0.186	0.033	0.000	0.120	0.251
Workplace struggles —> Burnout —> Turnover intention	0.084	0.021	0.000	0.040	0.126
Clients-related challenges —> Burnout—> Turnover intention	0.093	0.025	0.000	0.044	0.143
Clients-related challenges —> Work engagement —> Turnover intention	0.098	0.028	0.001	0.014	0.212
Fieldwork-related difficulties —> Burnout —> Turnover intention	0.038	0.048	0.003	−0.063	0.125

In the SEM, there was a direct effect of poor working environment on turnover intention (β = 0.540) and burnout (β = 0.296), and engagement (β = −0.660). Results also showed that working environment had an indirect effect on turnover intention (β = 0.186) through burnout and work engagement as mediators (total β = 0.726). The direct effect of workplace struggles on turnover intention (β = 0.313) was positive and significant, the indirect effect is also positive (β = 0.084), but weak (total β = 0.396), and a direct effect on burnout (β = 0.104) and engagement (β = −0.336). Clients-related challenges had a direct effect on turnover intention (β = 0.189), burnout (β = 0.145), and work engagement (β = −0.341), and an indirect effect on turnover intention (β = 0.093) through burnout and work engagement (total β = 0.193). Finally, fieldwork-related difficulties weren't significantly related to turnover but had a direct effect on burnout (β = 0.160), and engagement (β = −0.142), furthermore also an indirect effect on turnover intention through burnout (β = 0.032).

Additionally, associations were observed between turnover intention and the two mediating variables. A higher level of burnout was positively associated with increased turnover intention (β = 0.238). Conversely, turnover intention was negatively influenced by work engagement (β = −0.174), with higher work engagement linked to lower turnover intention. Furthermore, there was a significant relationship between the two mediating variables—work engagement and burnout (β = −0.234), indicating that higher work engagement is associated with lower levels of burnout.

### 3.4 Hierarchical multiple linear regression analysis of the factors related to social workers' turnover intention

Hierarchical multiple regression modeling was conducted with TIS as the dependent variable to examine turnover intention and relative influencing factors. A five-step process was followed. In the first regression model (Model 1), the sociodemographic factors were entered as independent variables. The second regression model (Model 2) included work-related characteristics. The third model (Model 3) incorporated the three subscales of working conditions as additional independent variables. This block should help reveal how general and specific work conditions influence turnover intentions, beyond the more static sociodemographic and job's inherent characteristics. Burnout dimensions (exhaustion and disengagement) were added into the fourth model (Model 4) to show the impact of psychological strain on turnover once other factors are controlled for. Finally, work engagement factors like vigor, dedication, and absorption were entered into the fifth regression model (Model 5) to assess how positive work attitudes contribute to mobility intention over and above burnout and other variables.

As shown in Table 3, Model 1 included five independent demographical characteristics: gender, age, marital status, subjective income, and education background. The sociodemographic variables could clarify intention to exit [*F*_(df)_ = 6.521 (6,655); *p* = 0.000)], although only two variables to reach significance were age and subjective income (model explanatory variance = 5.6%). The age negatively influenced turnover intention (β = −0.01, *p* = 0.007): older social workers are less likely to intention to exit. The subjective income status also significantly increased the risk of turnover intention: a significantly higher incidence of intention to exit can be expected among those living with income difficulties (β = 0.48, *p* = 0.000) than among those having a comfortable life as a result of their income situation.

**Table 3:** Determinants of turnover intention by hierarchical regression analysis.

**Variables**	**Model 1**		**Model 2**		**Model 3**		**Model 4**		**Model 5**	
	β	***P*** **-value**	β	***P*** **-value**	β	***P*** **-value**	β	***P*** **-value**	β	***P-*** **value**
**Sociodemographic variables**										
Gender	0.003 (0.109)	0.972	0.003 (0.109)	0.974	0.041 (0.086)	0.630	0.071 (0.082)	0.381	0.061 (0.079)	0.436
Age	−0.010 (0.004)	**0.007**	−0.013 (0.004)	**0.003**	−0.011 (0.003)	**0.002**	−0.007 (0.003)	**0.024**	−0.008 (0.003)	**0.049**
Marital status	−0.024 (0.085)	0.772	−0.012 (0.084)	0.883	−0.035 (0.066)	0.593	−0.023 (0.063)	0.707	−0.042 (0.061)	0.490
**Subjective income (Ref: they can easily live off their income)**										
Very hard and hard	0.483 (0.096)	**0.000**	0.446 (0.096)	**0.000**	0.034 (0.096)	0.666	0.066 (0.075)	0.519	0.048 (0.069)	0.484
Relatively easy	0.140 (0.096)	0.145	0.114 (0.095)	0.233	0.028 (0.075)	0.711	0.042 (0.071)	0.698	0.024 (0.072)	0.740
Social work degree	0.219 (0.092)	0.054	0.074 (0.071)	0.305	0.083 (0.078)	0.289	0.103 (0.074)	0.163	0.074 (0.071)	0.160
**Work–related characteristics**										
Social work experience			−0.018 (0.005)	**0.047**	−0.022 (0.004)	**0.048**	−0.018 (0.003)	**0.046**	−0.022 (0.004)	**0.041**
Job position			0.194 (0.090)	**0.029**	0.121 (0.072)	**0.039**	0.117 (0.068)	**0.040**	0.125 (0.066)	**0.035**
**Type of specialist area of care (Ref: social basic services)**										
Family and child welfare			0.158 (0.094)	0.076	0.094 (0.076)	0.217	0.126 (0.072)	0.081	0.108 (0.069)	0.121
Child protection			0.309 (0.146)	**0.006**	0.206 (0.116)	**0.036**	0.175 (0.110)	**0.014**	0.314 (0.106)	**0.009**
Specialized social care			0.003 (0.100)	0.872	0.024 (0.080)	0.258	0.003 (0.075)	0.114	0.017 (0.073)	0.112
Atypical work schedule			0.263 (0.130)	**0.046**	0.145 (0.105)	**0.047**	0.152 (0.099)	**0.046**	0.146 (0.097)	**0.046**
Overtime			0.175 (0.081)	**0.031**	0.169 (0.064)	**0.042**	0.168 (0.061)	**0.037**	0.164 (0.059)	**0.031**
**Working conditions subscales**										
Poor working environment					0.565 (0.031)	**0.000**	0.470 (0.031)	**0.000**	0.419 (0.031)	**0.000**
Workplace struggles					0.361 (0.035)	**0.000**	0.238 (0.033)	**0.000**	0.221 (0.032)	**0.003**
Clients-related challenges					0.085 (0.036)	**0.020**	0.084 (0.034)	**0.015**	0.082 (0.033)	**0.014**
Fieldwork-related difficulties					0.068 (0.035)	0.056	0.057 (0.034)	0.094	0.047 (0.033)	0.154
**Burnout subscales**										
Exhaustion							0.230 (0.030)	**0.000**	0.212 (0.030)	**0.000**
Disengagement							0.171 (0.031)	**0.000**	0.125 (0.032)	**0.014**
**Work engagement subscales**										
Vigor									−0.175 (0.064)	**0.007**
Dedication									−0.172 (0.066)	**0.010**
Absorption									−0.108 (0.067)	0.107
*N*	662		661		661		661		661	
*R* ^2^	0.056		0.092		0.436		0.498		0.536	
Adjusted *R*^2^	0.047		0.073		0.421		0.483		0.519	
*R*^2^ change	0.056		0.036		0.344		0.062		0.038	
	*p* = 0.000		*p* = 0.000		*p* = 0.000		*p* = 0.000		*p* = 0.000	
*F*(df)	6.52 (6.65)		5.03 (13.64)		29.24 (17.64)		33.53 (19.64)		33.44 (22.63)	
*F*(df) change			3.61 (7.64)		98.08 (4.64)		39.921 (2.64)		16.98 (3.63)	
			*p* = 0.001		*p* = 0.000		*p* = 0.000		*p* = 0.000	

The standard errors (SE) are reported in parentheses.

Ref, Reference Category. Significant P-values are highlighted in bold. Significance level was set at *P* < 0.05.

After controlling for sociodemographic variables and introducing work-related characteristics in Model 2, the results showed a statistically significant correlation to clarify the intention to exit with 9.2% variability [*F*_(df)_ = 5.037 (13,647); *p* = 0.001]. The explanatory variance increased by 3.6%, with work experience, job position, type of specialist area, atypical work schedule, and overtime both reaching significant effects. The effect of child protection as institutional type was the highest; β = 0.30 (*p* = 0.006), indicating that social professionals in child protection had higher turnover intention than those working in social basic services. Atypical work schedule (β = 0.26, *p* = 0.046) and overtime (β = 0.17, *p* = 0.031) were also significant and positive in the model of turnover intention, indicating that regular night and/or weekend work schedules and significant overtime (more than 10 h per week) increase the intention to quit the workplace. The frontline job position had also significant effect (β = 0.19, *p* = 0.029) in Model 2: frontline staff are more likely to have turnover intentions than workers in managerial positions. In addition, social professionals who have been working in the social sphere for less time are less likely to intend to quit their job (β = −0.018, *p* = 0.047).

Model 3, with working conditions subscales, including all independent characteristics, was quite better, with an R2 of 0.43 [*F*_(df)_ = 29.242 (17,643)], thus 43.6% of the variance had been accounted for. The change in R2 was highly significant [*F*_(df)_ = 98.084 (4,643); *p* = 0.000)], specifying that the additive working conditions indicators increased explanatory variance by 34.4%. Since our models were built step by step, it can be checked whether the effect of previously included sociodemographic and work-related variables changes after the inclusion of working conditions subscales. It was found that although the subjective income was found to be a statistically significant variable in the first and second models, it hadn't impact on the third model. Among the general and occupational-specific working conditions factors, the predictive power of poor working environment was the highest (β = 0.56, *p* = 0.000), indicating inadequate working environment was significantly associated with higher mobility intentions. Additionally, workplace struggles (β = 0.36, *p* = 0.000), and clients-related challenges (β = 0.08, *p* = 0.020) were as a positive predictors of turnover intention, while difficulties related to fieldwork had no significant effect on exit intention.

In the fourth model (Model 4), the in addition to indicators of sociodemographic, job's inherent characteristics and working conditions subscales, the subscales of burnout syndrome were also included. The inclusion of the burnout subscales did not change the significant effect of the already included variables in the models. The Model 4 significantly improved with adjusted R-square 0.498, i.e., the model improved by 6.2% (*p* = 0.000). The ANOVA table of the fourth model was also statistically significant [*F*_(df)_ = 33.534 (19,641); *p* = 0.000)]. With regard to the β coefficients of burnout dimensions, both exhaustion (β = 0.23, *p* = 0.000) and disengagement (β = 0.17, *p* = 0.000) increased the turnover intention.

Finally, the impact of work engagement subscales was analyzed in fifth model (Model 5). We found that when work engagement subscales entered, the model improved at 3.7% for the adjusted R-square 0.536. The ANOVA result was statistically significant [*F*_(df)_ = 33.444 (22,663); *p* = 0.000] for the Model 5. In terms of the subscales of work engagement, vigor (β = −0.17, *p* = 0.007), and dedication (β = −0.17, *p* = 0.010) proved to be negative predictors of intention to leave the workplace among social workers, but absorption had no significant effect on intention to exit.

The hierarchical regression analysis revealed that the working conditions subscales, such as poor working environment, workplace struggles, and clients-related challenges (34.4% contribution) were the most critical predictors of intention to exit, which appeared to be strong predictors of turnover intention. Both subscales of burnout syndrome, exhaustion and disengagement, played essential—although less than working conditions indicators—roles in intention to exit at 6.2%. Work engagement, work-related characteristics and age as sociodemographic variable also had impacts on turnover intention, but with a contribution of <6%.

## 4 Discussion

In this study, we investigated the interrelationship between general and occupational-specific working conditions, burnout, work engagement, and turnover intention, among Hungarian social workers. Overall, social professionals perceive financial and moral appreciation as the least satisfactory dimensions of the working environment. They also face challenges such as excessive administration and difficulties due to frequently changing rules and regulations, which contribute to inefficiency. Additionally, dealing with the specific culture of clients and cooperation issues emerged as significant challenges in their everyday work. A high proportion of social workers (67%) reported experiencing exhaustion, while a smaller proportion reported disillusionment (55%). Despite these challenges, their commitment to work remained relatively high, with only one-tenth reporting low engagement. However, one-third expressed strong intentions to quit.

Our results revealed that poor working environment, workplace struggles, and clients-related challenges significantly predicted turnover intention. These general and occupational-specific working conditions indicators increased explanatory power by 34% in the hierarchical multiple regression model, indicating that these are the most critical predictors of intention to exit. Among the working conditions factors, the strongest correlation was observed between the poor working environment factor and mobility intention. This suggests that social workers who feel demotivated within the organization—experiencing limited opportunities for advancement, low salaries, lack of financial or moral respect, restricted development opportunities, limited participation in decision-making, an unsupportive work atmosphere, and poor/inappropriate work environment—are at higher risk for turnover intention. These results underscore the privotal role of workplace and organizational factors in driving mobility intentions (Demerouti et al., 2001; Kim and Stoner, 2008; Harrington et al., 2005; Huang et al., 2003). Furthermore, the SEM analysis revealed that the poor working environment also had a significant indirect effect on turnover intention mediated by burnout and work engagement. The direct effect of workplace struggles and clients-related challenges on turnover intentions was smaller compared to the impact of the working environment. For instance, difficulties in cooperation with clients, managing cultural differences, real-life situation management, and associated role conflicts all contributed to increased turnover intention among professionals. These findings align with previous research showing that role conflicts (Hongfei et al., 2023; Hoseini et al., 2021) and family-workplace discrepancies (Roberts et al., 2014) significantly influence intention to leave the workplace. These correlations also occurred through indirect effects: both workplace struggles and clients-related problems had a significant indirect effect on social workers' turnover intention by burnout and work engagement. While the increased volume of fieldwork problems did not directly affect social workers' turnover intentions, it did indirectly impact the intention to quit by exacerbating burnout.

In addition to mediating between working conditions and turnover intention, burnout significantly negative impacts social workers' intentions to quit. Previous research has confirmed that burnout affects social workers' turnover (Kim and Stoner, 2008; Nilsen et al., 2023; Zhang et al., 2022). However, many studies have focused primarily on emotional exhaustion and have not included disengagement in their analyses. By including both dimensions in our analysis, we found that both exhaustion and disengagement predicted turnover intent, with exhaustion having a stronger effect on turnover than disengagement.

In professions dedicated to assisting marginalized social groups, it's evident that the helpers themselves require significant support, as the appearance of an intention to quit indicates job dissatisfaction. Among the deficiencies in this support, low wages and excessive workload stand out, necessitating administrative government and maintenance measures. The mental strain resulting from the nature of the work, evidenced by the notably high incidence of burnout, also demands institutional-level solution. This includes continuously strengthening social workers' problem-management competences through initiatives such as supervision or other organized professional assistance, aiding in a better understanding of clients' social and life situations.

Several studies have established a significant negative relationship between work engagement and turnover intention (Bothma and Roodt, 2012; Ivanovic and Maricic, 2020; Ravalier, 2018). Our results also demonstrated that social workers with higher levels of engagement had significantly lower mobility intentions. Furthermore, findings from linear multiple regression analysis indicated that two out of the three dimensions of engagement– vigor and dedication–significantly influenced the intention to quit. Dedication exhibited a stronger effect on turnover than vigor.

Finally, this study confirmed that several other work-related and sociodemographic factors significantly impacted intention to exit. Our results showed that job position had significant direct effects on turnover intent. Consistent with previous research (Cho and Song, 2017; Wang et al., 2019), frontline positions, characterized by frequent direct and stressful interactions with clients, emerged as important predictors of turnover intention. Furthermore, the study confirmed a direct and negative relationship between turnover intention: individuals with less experience in social care are more likely to plan job changes (Gupta and Shaheen, 2017). Institutional professional support, as mentioned earlier, may be particularly crucial for younger, novice professionals, as they are more prone to career changes, resulting in loss of previously acquired social professional knowledge from the system.

Another remarkable result of our analysis is that child protection, as a field within social work, significantly influenced turnover intention. Social workers in child protection were more likely to intend to quit their job compared to those in social basic services used as a reference. This finding is consistent with previous research (Conrad and Kellar-Guenther, 2006).

Additionally, confirming previous research findings (Blytt et al., 2022; Jiang et al., 2022; Johnson and Lipscomb, 2006), unconventional work schedules and overtime also significantly increased turnover intentions. Among the sociodemographic variables examined in this study, only age had a significant negative effect on intention to quit (Kim and Kao, 2014; Su, 2020). Presumably, early in one's career, job insecurity, lower wages and lack of experience act as stressors that increase the intention to exit. Several other studies also suggest that demographic factors such as gender or education have minimal influence on turnover intent (Kim and Kao, 2014; Pugh, 2016).

The findings of this study could serve as a starting point for future research aimed at a deeper understanding of the issue of labor turnover in the social services sector. Additionally, our results can offer guidance for social care organizations develop more targeted measures to retain their workforce.

In conclusion, of our analysis emphasizes the necessity of creating conducive organizational conditions and incentives to mitigate the intention to quit. Furthermore, it highlights the importance of preparing for the content elements of work, real-life situations, and related conflicts and difficulties. Additionally, there is a need to establish continuous professional support institutions and foster cooperation.

### 4.1 Limitation

To the best of our knowledge, this is the first study to examine the association between profession-specific working conditions, burnout, work engagement and turnover intention among social workers. Despite its strengths, our research also has several limitations. Firstly, due to its cross-sectional nature, it is not possible to draw conclusions about causal relationships. Secondly, the data collected relied on self-reporting, which is subject to well-established method biases. Thirdly, current study only focused turnover intention, not actual turnover behavior.

## 5 Conclusion

This study examined the impact of general and occupational-specific working conditions, burnout, and work engagement on social professionals' turnover intent to identify contributing factors to their intention to quit. Our findings indicated that poor working environment, workplace struggles, and challenges related to clients played crucial roles in social workers' turnover intention. Additionally, the results showed that these aspects of working conditions not only directly and significantly increased professionals' turnover intention, but also indirectly influences it through burnout and work engagement as mediators. This suggests that both general working conditions and profession-specific factors warrant attention due to their roles in mitigating social workers' intention to quit.

To motivate and retain social workers, targeted strategies should be implemented. Given that profession-specific factors—such as challenges stemming from clients' difficult life situations, difficulties in client cooperation, cultural disparities, and fieldwork-related challenges—are significant factors in professionals' decisions to leave their jobs or careers, we recommend integrating content elements addressing real-life situations, conflicts, and difficulties into the social work curriculum within educational programs in Hungary. This would help better prepare social workers mentally to handle the complexities they encounter in their field.

## Data Availability

The raw data supporting the conclusions of this article will be made available by the authors, without undue reservation.
